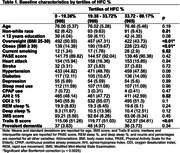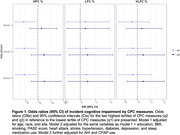# Cardiopulmonary coupling measures of sleep stability and risk of cognitive impairment in community‐based older men

**DOI:** 10.1002/alz.085901

**Published:** 2025-01-09

**Authors:** Sasha Milton, Haoqi Sun, Katie L Stone, Kristine Yaffe, Brandon Westover, Robert J Thomas, Yue Leng

**Affiliations:** ^1^ University of California, San Francisco, San Francisco, CA USA; ^2^ Massachusetts General Hospital, Boston, MA USA; ^3^ Beth Israel Deaconess Medical Center, Boston, MA USA; ^4^ California Pacific Medical Center Research Institute, San Francisco, CA USA; ^5^ San Francisco Veterans Affairs Health Care System, San Francisco, CA USA; ^6^ Departments of Psychiatry and Behavioral Sciences, Neurology, and Epidemiology, University of California San Francisco, San Francisco, CA USA

## Abstract

**Background:**

Poor sleep health is associated with cognitive impairment (CI) in older adults. The cardiopulmonary coupling (CPC) sleep spectrogram, which estimates the sleep state modulated synchronization between heart rate variability and respiration, is an increasingly recognized measure of sleep stability. However, little is known about the longitudinal association between CPC measures of sleep stability and risk of incident CI.

**Method:**

Our sample comprised 2628 men (mean [SD] age = 76.0 [5.3] years, all aged ≥65 years) without CI who had their sleep assessed using polysomnography at baseline (2003‐05) and were followed until 2016 for incident CI as part of the Osteoporotic Fractures in Men (MrOS) study. Electrocardiogram (ECG)‐derived CPC metrics included the percentage of sleep time spent with high frequency coupling (HFC%, 0.1‐0.4Hz, stable sleep), low frequency coupling (LFC%, 0.01‐0.1Hz, unstable sleep), and very low frequency coupling (VLFC%, <0.01Hz, an estimation of rapid eye movement sleep and wakefulness). Incident CI (encoded as a binary variable) was defined as self‐reported dementia diagnosis, dementia medication use, Modified Mini‐Mental State Examination (3MS) score <80, or a decline in 3MS score from baseline to any visit of ≥1.5 standard deviations below the mean. Multivariable logistic regression was used to assess the associations between tertiles of CPC metrics and incident CI.

**Result:**

A total of 419 men developed CI during a mean follow‐up of 10.8 years. After adjustment for covariates including demographics, lifestyle, comorbidities, medication use, and cognition at baseline, men in the middle tertile of HFC% were less likely to develop CI than men in the lowest tertile [OR (95% CI) = 0.73 (0.56,0.96)], while those in the highest tertile of HFC% had similar odds of incident CI [OR (95% CI) = 1.12 (0.87,1.45)] compared with the lowest tertile. Further adjustment for apnea‐hypopnea index (AHI) and use of continuous positive airway pressure (CPAP) did not appreciably alter these results. Neither LFC% nor VLFC% was associated with incident CI.

**Conclusion:**

Older men with intermediate levels of stable sleep had the lowest risk of developing CI over 11 years. Further research is needed to understand the mechanisms linking CPC measures of sleep stability to cognitive aging.